# Frequent amplification of *ORAOV1* gene in esophageal squamous cell cancer promotes an aggressive phenotype via proline metabolism and ROS production

**DOI:** 10.18632/oncotarget.1561

**Published:** 2013-12-30

**Authors:** Yosuke Togashi, Tokuzo Arao, Hiroaki Kato, Kazuko Matsumoto, Masato Terashima, Hidetoshi Hayashi, Marco A de Velasco, Yoshihiko Fujita, Hideharu Kimura, Takushi Yasuda, Hitoshi Shiozaki, Kazuto Nishio

**Affiliations:** ^1^ Department of Genome Biology, Kinki University Faculty of Medicine, Osaka-Sayama, Osaka, Japan; ^2^ Department of Surgery, Kinki University Faculty of Medicine, Osaka-Sayama, Osaka, Japan

**Keywords:** oral cancer overexpressed 1, esophageal squamous cell cancer, pyrroline-5-carboxylate reductase, proline metabolism, reactive oxygen species

## Abstract

Chromosomal band 11q13 seems to be one of the most frequently amplified lesions in human cancer, including esophageal squamous cell cancer (ESCC). The oral cancer overexpressed 1 (*ORAOV1*) gene has been identified within this region, but its detailed biological function in human ESCC remains largely unclear. In our clinical samples of stage III ESCC, *ORAOV1* amplification was observed in 49 of 94 cases (53%). *ORAOV1* amplification was significantly associated with a poorly differentiated histology and tumors located in the upper or middle esophagus. Patients with *ORAOV1* amplification tended to have a shorter survival period, although the difference was not significant. To investigate the function of *ORAOV1*, we created *ORAOV1*-overexpressed ESCC cell lines that exhibited increased cellular proliferation and colony formation, compared with *in vitro* controls. *In vivo, ORAOV1*-overexpressed cells exhibited a significantly increased tumorigenicity and a significantly larger tumor volume and poorer differentiation than controls. The peptide mass fingerprinting technique demonstrated that ORAOV1 bound to pyrroline-5-carboxylate reductase (PYCR), which is associated with proline metabolism and reactive oxygen species (ROS) production. Then, *ORAOV1*-overexpressed cell lines were resistant to stress treatment, which was cancelled by *PYCR*-knockdown. In addition, the *ORAOV1*-overexpressed cell line had a higher intracellular proline concentration and a lower ROS level. Our findings indicate that the *ORAOV1* gene is frequently amplified in ESCC, enhances tumorigenicity and tumor growth, and is associated with a poorly differentiated tumor histology via proline metabolism and ROS production. ORAOV1 could be a novel target for the treatment of ESCC.

## INTRODUCTION

Among the malignancies of the gastrointestinal tract, esophageal cancer is associated with a poor prognosis despite improvements in surgical techniques, chemotherapy, and chemoradiotherapy [1, 2]. Neoadjuvant chemo-radiation followed by surgery remains the standard of care for operable disease. Definitive chemo-radiation can be considered for locally advanced tumors, while platinum-based combination chemotherapy is preferable in a first-line metastatic setting. Recently, *HER2-*, *EGFR-*, and *VEGF*-targeted agents have been extensively investigated as single agents or in combination with chemotherapy. Furthermore, several new targets, including *mTOR*- and *MET*-targeted agents, are also being explored [2]. However, none of the molecular-targeted therapies developed to date have enabled a demonstrated improvement in the survival of patients with esophageal cancer. Therefore, the search for novel targeted molecules must be continued.

Chromosomal band 11q13 seems to be one of the most frequently amplified lesions in human cancer, including esophageal squamous cell cancer (ESCC), and can be associated with an advanced stage and poor prognosis [3-6]. Several known cancer-related genes, such as *CCND1*, *FGF4*, *FGF3*, and *CTTN*, have been mapped to the 11q13 chromosome region [6]. The oral cancer overexpressed 1 (*ORAOV1*) gene has also been identified within this region and is presumably a candidate treatment target for oral squamous cell cancer [7, 8]. Several studies have shown that *ORAOV1* is related to the cell cycle, apoptosis and angiogenesis [8, 9]. Furthermore, a recent study has demonstrated that a yeast orthologue of the ORAOV1 protein is related to reactive oxygen species (ROS) production. However, the detailed biological functions of *ORAOV1* gene in human cancer remain unclear [10]. In addition, only one report showing a relationship between the *ORAOV1* gene and ESCC has been published [11]. In the present study, we investigated the relationship between *ORAOV1* amplification and the clinicopathological features of patients with ESCC and the detailed biological functions of the *ORAOV1* gene.

## RESULTS

### Tissue distribution of *ORAOV1* mRNA in normal human tissue and several human cell lines

**Figure 1:**
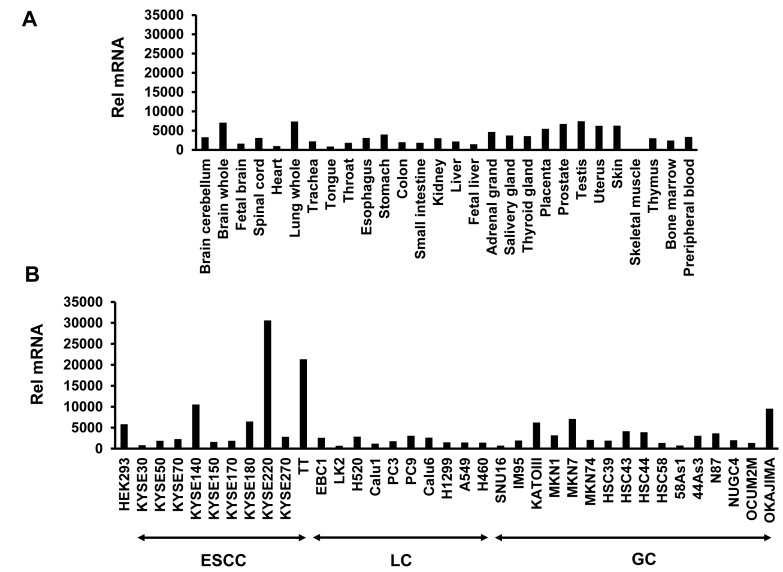
Tissue distribution of *ORAOV1* mRNA expression The *ORAOV1*mRNA expression levels were determined using a real-time RT PCR analysis. A, Human normal tissue. No high levels of *ORAOV1* mRNA expression were found, even in the tongue, throat, and esophagus. B, Human cancer and HEK293 cell lines. Several ESCC cell lines (especially the KYSE220 and T.T cell lines) had a very high *ORAOV1* mRNA expression level. ESCC, esophageal squamous cell cancer; LC, lung cancer; GC, gastric cancer; Rel mRNA, normalized mRNA expression levels (*ORAOV1/GAPD* × 10^6^).

To examine the tissue distribution of *ORAOV1* mRNA, we performed real-time reverse transcription PCR (RT-PCR) for normal human tissues. No high expression levels of *ORAOV1* mRNA were found, even in the tongue, throat, or esophagus (Figure 1A). *ORAOV1* expression was also examined in 37 human cell lines. A very high *ORAOV1* mRNA expression level was observed in several ESCC cell lines (especially, KYSE220 and T.T), whereas the levels in lung cancer, including squamous cell cancer and gastric cancer, were not so high (Figure 1B).

### *ORAOV1* gene amplification in ESCC cell lines and surgical specimens

**Figure 2:**
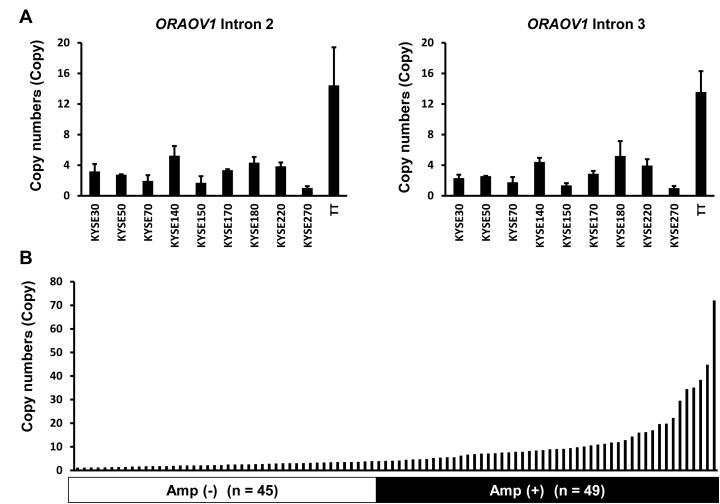
The *ORAOV1* gene was amplified in ESCC cell lines and surgical specimens A, Evaluation of DNA copy number assay using ESCC cell lines. A TaqMan copy number assay was performed to determine the copy number using specific primers for genomic loci of the *ORAOV1* gene against DNA samples. The experiment was performed in triplicate. Using a cut-off of 4 copies, the numbers were 0.98-3.3 copies in the non-amplified cell lines; however, the numbers in the *ORAOV1*-amplified cell lines were 4.2-14.4 copies (KYSE140, KYSE180, KYSE220, and T.T). Both primers that were used (Hs03772057_cn [intron >2] and Hs03793932_cn [intron 3]) produced very similar results (R^2^ = 0.98). Columns, mean of independent triplicate experiments; bars, SD. B, Amplification of *ORAOV1* gene in surgical specimens of ESCC. *ORAOV1* amplification was evaluated using Hs03772057_cn (intron 2) in 94 FFPE samples of ESCC specimens. *ORAOV1* amplification producing more than 4 copies was observed in 49 cases, with a frequency of 53%.

To develop a high-throughput method for detecting *ORAOV1* amplification in a clinical setting, we verified a real-time PCR-based detection method, the TaqMan Copy Number Assay. Using a cut off of 4 copies, the number was 0.98-3.3 copies in the non-amplified cell lines; however, the number in the *ORAOV1*-amplified cell lines was 4.2-14.4 copies (KYSE140, KYSE180, KYSE220, and T.T). In addition, both primers that we used (intron 2 and intron 3) produced very similar results (R^2^ = 0.98, Figure 2A). These results suggested that a DNA copy number assay for the *ORAOV1* gene was a sensitive and reproducible method. Next, *ORAOV1* amplification was evaluated using Hs03772057_cn (intron 2) in 94 FFPE samples of stage III ESCC specimens. *ORAOV1* amplification of more than 4 copies was observed in 49 cases, with a frequency of 53% (Figure 2B).

### Clinicopathological features of *ORAOV1*-amplified ESCC

**Figure 3:**
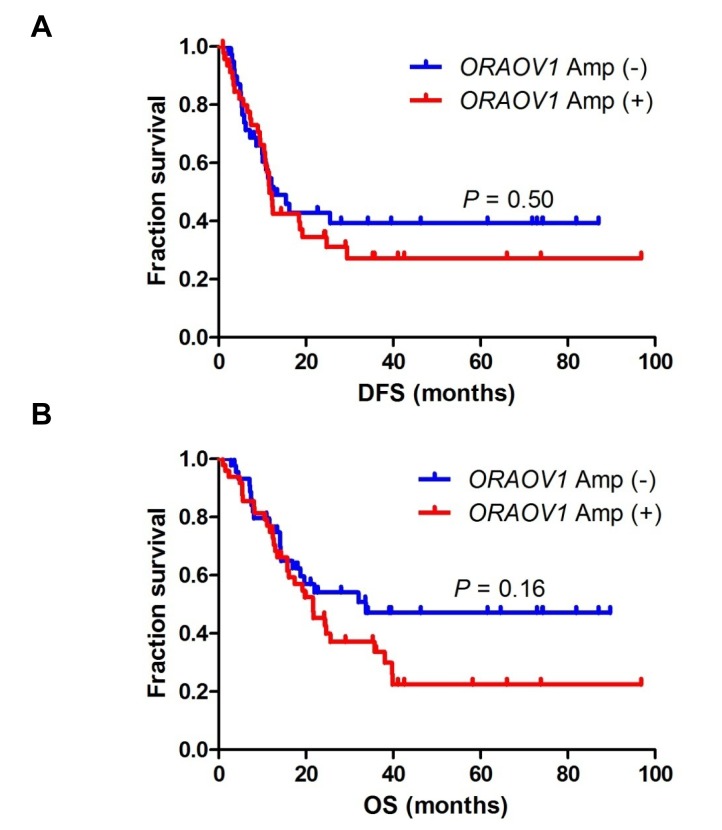
DFS and OS after surgery in patients with stage III ESCC A, DFS. Patients with *ORAOV1* amplification tended to have a shorter DFS compared with patients without amplification, although the difference was not significant (median DFS, 11.6 vs. 12.6 months, *P* = 0.50). B, OS. Patients with *ORAOV1* gene amplification tended to have a shorter OS compared with patients without amplification, although the difference was not significant (median OS, 21.6 vs. 33.7 months, *P* = 0.16).

We evaluated the clinicopathological features of patients with stage III ESCC according to the *ORAOV1* amplification status. No significant differences in age, sex, or disease stage were seen between patients classified according to the *ORAOV1* amplification status, whereas the histology and tumor location were significantly associated with *ORAOV1* amplification (Table 1). Specifically, patients with *ORAOV1* amplification tended to have poorly differentiated tumors in the upper or middle region of the esophagus. In addition, we examined the prognostic significance of *ORAOV1* amplification. Patients with *ORAOV1* amplification tended to have a shorter disease-free survival (DFS) and overall survival (OS) after surgery, compared with patients without amplification, although the differences were not significant (median DFS, 11.6 vs. 12.6 months, *P* = 0.50, and median OS, 21.6 vs. 33.7 months, *P* = 0.16, respectively) (Figure 3A and B).

**Table 1:** Associations between patient characteristics and ORAOV1 gene amplification (n = 94)

Patients characteristics	*ORAOV1*		*P*-values
	Amp (−)	Amp (+)	
Number	45	49	
Age (years)			
<70	33	34	0.82
>70	12	15	
Sex			
Male	39	43	1.00
Female	6	6	
Location			
Ut or Mt	20	35	0.012*
Lt or Ae	25	14	
Tumor differentiation			
Well or moderate	41	36	0.033*
Poor	4	13	
pStage			
IIIA	17	26	0.15
IIIB or IIIC	28	23	
Lymphatic invasion			
0	9	8	0.79
1 or 2	36	41	
Vascular invasion			
0	33	38	0.81
1	12	11	
Residual cancer			
0	7	5	0.54
1 or 2	38	44	

Abbreviations: Amp, amplification; Ut, upper thoracic esophagus; Mt, middle thoracic esophagus; Lt, lower thoracic esophagus; Ae, abdominal esophagus.

### Overexpression of *ORAOV1* gene enhanced cellular growth and colony formation, but not cellular attachment and migration

**Figure 4:**
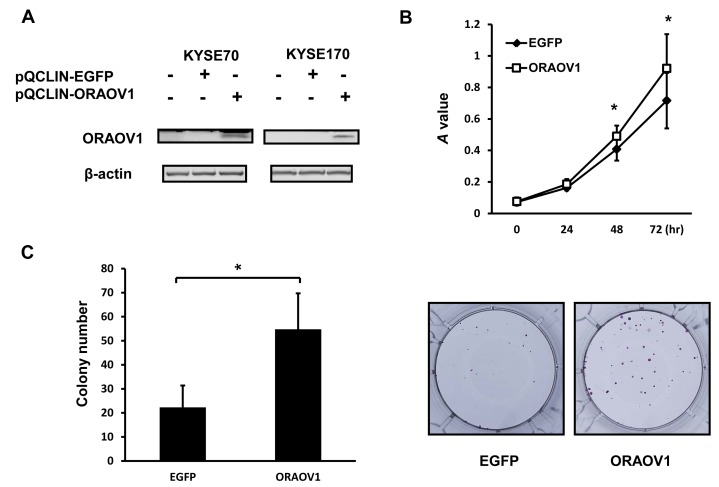
*ORAOV1*-overexpressed cell lines exhibited enhanced cellular growth and colony formation To investigate the biological function of the *ORAOV1* gene, the *EGFP* or *ORAOV1* gene was retrovirally introduced into the KYSE70 and KYSE170 cell lines (KYSE70-pQCLIN-EGFP or KYSE70-pQCLIN-ORAOV1 and KYSE170-pQCLIN-EGFP or KYSE170-pQCLIN-ORAOV1, respectively). A, Immunoblotting analyses of the KYSE70 and KYSE170 cell lines. The overexpression of the *ORAOV1* gene was confirmed using western blot analyses with a specific antibody for ORAOV1. β-actin was used as an internal control. B, Cellular growth of the KYSE70 transfectant cell lines. A total of 2,000 cells of each cell line were seeded into 96-well plates and were evaluated after 0, 24, 48, and 72 hours using an MTT assay. The experiment was performed in triplicate. The KYSE70-pQCLIN-ORAOV1 cell line showed increased cellular proliferation, compared with the control (KYSE70-pQCLIN-EGFP) (0 h, *P* = 0.55; 24 h, *P* = 0.15; 48 h, *P* = 0.0011*; 72 h, *P* = 0.013*). Lines, mean of independent triplicate experiments; bars, SD; *P < 0.05. C, Colony formation in KYSE70 transfectant cell lines. A total of 1,000 cells of each cell line were seeded into 6-well plates. After 2 weeks, the cells were stained with crystal violet and the number of colonies was counted. The experiment was performed in triplicate. The KYSE70-pQCLIN-ORAOV1 cell line showed increased colony formation, compared with the control (KYSE70-pQCLIN-EGFP) (EGFP: 22.33 ± 9.06 vs. ORAOV1: 54.78 ± 14.92, *P* = 0.015*). Columns, mean of independent triplicate experiments; bars, SD; *P < 0.05.

To elucidate the biological function of the *ORAOV1* gene, the *EGFP* or *ORAOV1* gene was retrovirally introduced into the KYSE70 and KYSE170 cell lines. The stable cell lines were designated as KYSE70-pQCLIN-EGFP, KYSE70-pQCLIN-ORAOV1, KYSE170-pQCLIN-EGFP, and KYSE170-pQCLIN-ORAOV1, respectively (Figure 4A). We then performed cellular growth assays and colony formation assays using these cell lines. Both the KYSE70-pQCLIN-ORAOV1 and KYSE170-pQCLIN-ORAOV1 cell lines showed increased cellular proliferation and colony formation, compared with the controls *in vitro* (Figure 4B and C), indicating that the *ORAOV1* gene is involved in cellular growth and tumorigenicity.

We next performed adhesion, migration, and scratch assays. No difference in these assays was observed between the cell lines (Supplementary Figure 1). These results indicate that the *ORAOV1* gene is not involved in cellular motility.

### Overexpression of *ORAOV1* gene enhances tumorigenicity and tumor growth *in vivo*

**Figure 5:**
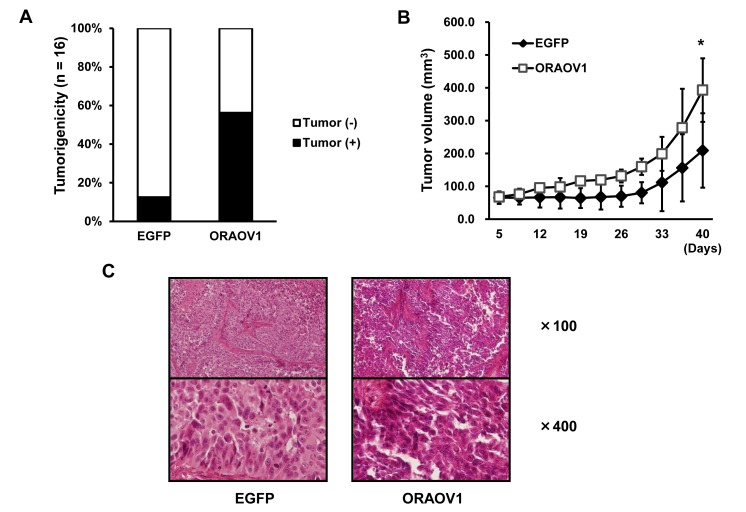
The *ORAOV1* gene enhanced tumorigenicity and tumor growth and was associated with a poorly differentiated tumor histology *in vivo* A, Tumorigenicity *in vivo*. To evaluate tumorigenicity, a suspension of 1 × 10^6^ KYSE70 transfectant cells (in 50 μL PBS) were subcutaneously inoculated into both flanks of nude mice (n = 8). Tumor formation was assessed every 2 or 3 days. The KYSE70-pQCLIN-ORAOV1 cell line exhibited a significantly elevated level of tumorigenesis *in vivo* (EGFP 2/16 vs. ORAOV1 9/16, *P* = 0.023*). B, Tumor growth *in vivo.* A total of 5 × 10^6^ KYSE70 transfectant cells (in 50 μL PBS) with 50 μL of Matrigel were subcutaneously inoculated into nude mice (n = 5). The tumor size was assessed every 2 to 3 days. The KYSE70-pQCLIN-ORAOV1 cell line produced a significantly larger tumor volume than the KYSE70-pQCLIN-EGFP cell line on day 40 (EGFP: 209 ± 113 vs. ORAOV1: 393 ± 97 mm^3^, *P* = 0.0041*). Lines, mean of 5 tumors; error bars, SD; *P < 0.05. C, Histology of the tumor. Hematoxylin-eosin staining showed that the KYSE70-pQCLIN-ORAOV1 cells exhibited a destroyed structure, an increased nucleus-cytoplasm ratio, and deeply stained nuclei, indicating a poorly differentiated tumor.

To examine the biological functions of the *ORAOV1* gene *in vivo*, we evaluated tumorigenicity and tumor growth using the KYSE70 transfectant cell lines. KYSE70-pQCLIN-ORAOV1 cells exhibited a significantly elevated level of tumorigenesis (EGFP: 2/16 vs. ORAOV1: 9/16, *P* = 0.023*), and a larger tumor volume than KYSE70-pQCLIN-EGFP on day 40 (EGFP: 209 ± 113 vs. ORAOV1: 393 ± 97 mm^3^, *P* = 0.0041*) (Figure 5A and B). In addition, KYSE70-pQCLIN-ORAOV1 cells produced poorly differentiated tumors (Figure 5C). These results indicate that the *ORAOV1* gene is involved in tumorigenesis and tumor growth, as seen *in vitro*, and is associated with a poorly differentiated histology.

### ORAOV1 binds to pyrroline-5-carboxylate reductase (PYCR)

**Figure 6:**
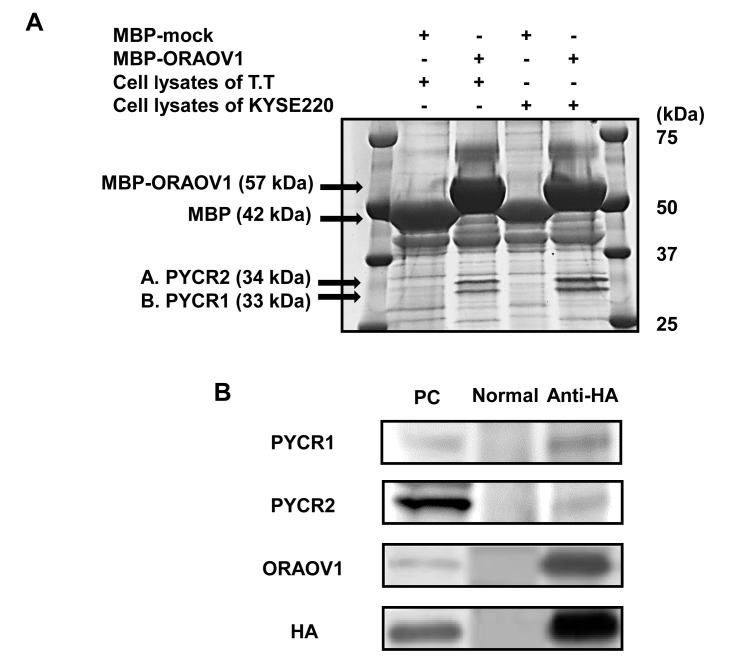
ORAOV1 binds to PYCR A, Silver-stained SDS-PAGE. Cell lyses of the T.T or KYSE220 cell lines were mixed with MBP-mock or MBP-ORAOV1 fusion protein. MBP-mock and MBP-ORAOV1with bound protein were absorbed by amylose resin. The samples were separated using SDS-PAGE, and the gel was stained with silver stain. The mass spectrometry data were acquired using a matrix-assisted laser desorption ionization/time-of-flight mass spectrometer. Protein identification based on the generated data was performed by searching a database using the online MASCOT search engine, revealing that band A and B represented PYCR2 and PYCR1, respectively. B, Immunoblot detection of ORAOV1 binding to PYCR1 and PYCR2. Cell lyses of HEK293-pcDNA-ORAOV1/HA/His (1 mg) were incubated with normal rat IgG (lane, normal) or anti-HA high-affinity rat antibody (lane, Anti-HA) for immunoprecipitation. Immunocomplexes were purified by incubation with protein G-agarose beads. As a positive control (lane, PC), cell lyses of the HEK293-pcDNA-ORAOV1/HA/His cell line (25 μg) were pulled down. PYCR1 and PYCR2 were confirmed using specific antibodies for PYCR1 and PYCR2, respectively. PC, positive control; normal, normal rat IgG; Anti-HA, anti-HA rat antibody.

To search for a protein that binds with ORAOV1, cell lyses of the T.T and KYSE220 cell lines were used, since the *ORAOV1* mRNA expression levels were very high in these cell lines. The peptide mass fingerprinting technique using maltose binding protein (MBP) fusion protein demonstrated that ORAOV1 bound to PYCR1 and PYCR2, which was confirmed by co-immunoprecipitation using the HEK293-pcDNA-ORAOV1/HA/His cell line (Figure 6A and B). These results suggest that ORAOV1 influences PYCR.

### *ORAOV1*-overexpressed cell lines were resistant to stress treatment via proline metabolism and ROS production

**Figure 7:**
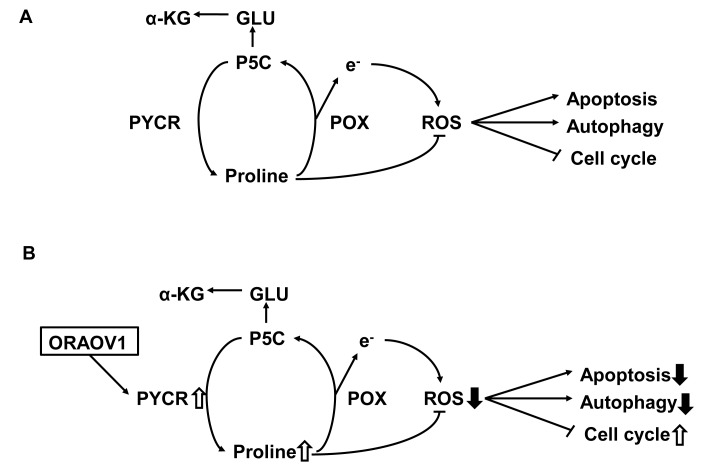
Diagram for the proposed effects of ORAOV1 on proline metabolism and ROS production A, Proline metabolic pathway during the normal state. Proline is oxidized to P5C by POX, and P5C is recycled back to proline by PYCR via redox transfers or is sequentially converted to glutamate and α-ketoglutarate. The metabolism of proline generates electrons to produce ROS and initiates a variety of downstream effects, including the blockade of the cell cycle, autophagy, and apoptosis. Despite its pro-oxidant effects, proline has been shown to protect mammalian cells against oxidative stress. Proline protection has been postulated to involve protein chaperoning, direct scavenging of ROS, and the upregulation of antioxidant enzymes. B, Proline metabolic pathway during *ORAOV1* overexpression. ORAOV1 influences PYCR activity; as a result, the intracellular proline level increases and ROS production decreases, thereby promoting tumor progression. GLU, glutamate; α-KG, α-ketoglutarate.

**Figure 8:**
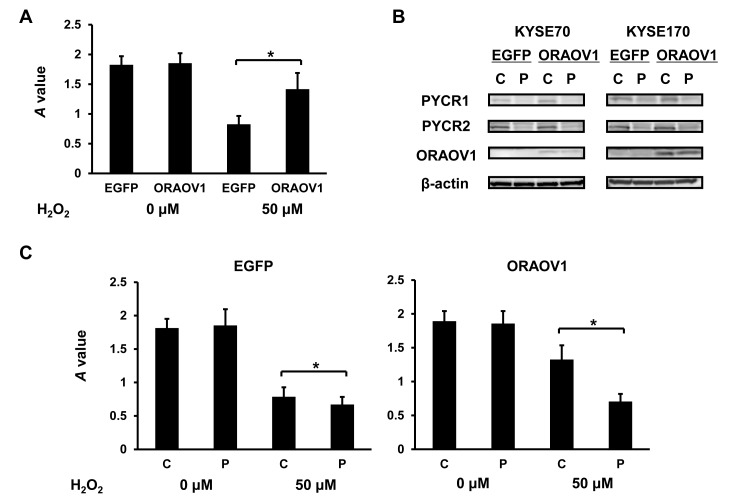
*ORAOV1*-overexpressed cell lines were resistant to stress treatment, which was cancelled by *PYCR*-knockdown A total of 20,000 cells from each cell line were plated onto 96-well plates. After 24 hours of incubation, the cells were treated for 12 hours with or without 50 μM of H_2_O_2_. Cell survival after stress treatment was estimated using the MTT assay. The experiment was performed in triplicate. A, Cell survival of the KYSE70 transfectant cell lines after H_2_O_2_ stress treatment. Compared with the control cells, more KYSE70-pQCLIN-ORAOV1 cells survived after 50 μM H_2_O_2_ treatment (without H_2_O_2_, *P* = 0.66; with H_2_O_2_, *P* = 0.039*). Columns, mean of independent triplicate experiments; bars, SD; *P < 0.05. B, Immunoblotting analyses of the KYSE70 and KYSE170 transfectant cell lines. *PYCR*-knockdown was confirmed in both cell lines. β-actin was used as an internal control. C, control (siRNA scramble); P, siRNA PYCR. C, Cell survival of the KYSE70 transfectant cell lines with or without *PYCR*-knockdown after H_2_O_2_ stress treatment. In the KYSE70-pQCLIN-EGFP cell line, *PYCR*-knockdown had a minimal influence on cell survival (*P* = 0.021*), whereas *PYCR*-knockdown greatly decreased cell survival after stress treatment in the KYSE70-pQCLIN-ORAOV1 cell line (*P* = 0.010*). C, control (siRNA scramble); P, siRNA PYCR.

Previous articles have shown that PYCR is associated with proline metabolism and ROS production (Figure 7A) [12-16]. Accordingly, resistance to stress treatment was next investigated. Cell survival after H_2_O_2_ stress treatment was significantly higher in the KYSE70-pQCLIN-ORAOV1 and KYSE170-pQCLIN-ORAOV1 cell lines than in the controls (Figure 8A). The stress-resistant effect was cancelled by *PYCR*-knockdown (Figure 8B and C).

**Figure 9:**
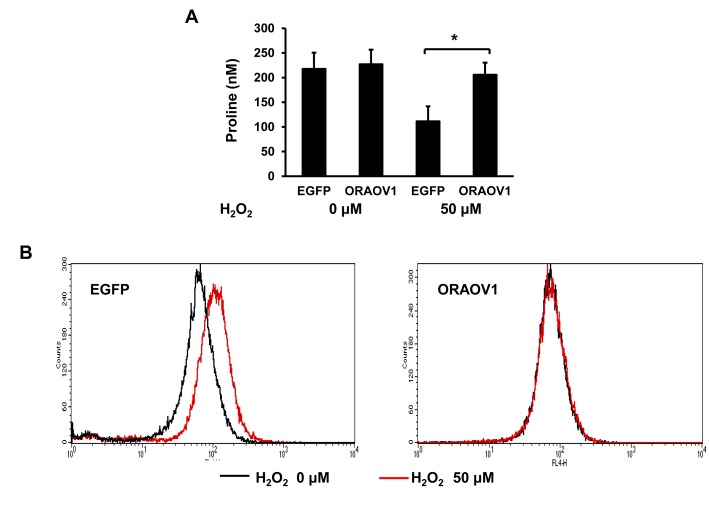
The *ORAOV1* gene was associated with proline metabolism and ROS production A, Intracellular proline concentrations of the KYSE70 transfectant cell lines. The proline concentrations were determined using high-performance liquid chromatography fluorescence detection using the pre-column derivatization procedure. The experiment was performed in triplicate. Intracellular proline concentrations after 50 μM of H_2_O_2_ stimulation were higher in the KYSE70-pQCLIN-ORAOV1 cell line than in the control (without H_2_O_2_, EGFP: 217.7 ± 32.8 vs. ORAOV1: 227.0 ± 29.6 nM, *P* = 0.78; with H_2_O_2_, EGFP: 111.4 ± 30.2 vs. ORAOV1: 205.7 ± 24.5 nM, *P* = 0.014*). Columns, mean of independent triplicate experiments; Bars, SD; *P < 0.05. B, ROS production of the KYSE70 transfectant cell lines. ROS production was measured using a flow cytometer and CellROX Deep Red Reagent. ROS production was lower in the KYSE70-pQCLIN-ORAOV1 cell line than in the control when the cell lines were stimulated with 50 μM of H_2_O_2_.

In addition, we measured the intracellular proline concentrations and ROS production. Intracellular proline concentration after stress treatment was significantly higher in the KYSE70-pQCLIN-ORAOV1 cell line than in the control (EGFP: 111.4 ± 30.2 vs. ORAOV1: 205.7 ± 24.5 nM, *P* = 0.014), and ROS production after stress treatment was lower in the KYSE70-pQCLIN-ORAOV1 cell line than in the control (Figure 9A and B). These results indicate that the *ORAOV1* gene is associated with resistance to stress treatment via proline metabolism and ROS production (Figure 7B).

## DISCUSSION

Chromosomal band 11q13 seems to be one of the most frequently amplified lesions in human cancer, including ESCC, and is associated with an advanced disease stage and a poor prognosis [3-6]. The *ORAOV1* gene has been identified within this region, but its detailed biological function in human cancer remains largely unclear. Our findings indicate that the *ORAOV1* gene enhances tumorigenicity and tumor growth and is associated with a poorly differentiated tumor histology in ESCC via proline metabolism and ROS production. To the best of our knowledge, this is the first report to show that the *ORAOV1* gene is associated with proline metabolism and ROS production in human cancer.

Proline is a multifunctional amino acid with important roles in primary carbon and nitrogen metabolism, osmotic and oxidative stress protection, protein chaperoning, cell signaling, programmed cell death, and nutrient adaption and survival. The metabolism of proline generates electrons to produce ROS and initiates a variety of downstream effects, including blockade of the cell cycle, autophagy, and apoptosis [12-16]. Proline is oxidized to pyrroline-5-carboxylate (P5C) by proline oxidase (POX), and P5C is recycled back to proline by PYCR via redox transfers or is sequentially converted to glutamate and α-ketoglutarate (Figure 7A). The effects of POX, as well as its decreased levels in tumors, support its role as a tumor suppressor via ROS production [16-18]. Despite its pro-oxidant effects, proline has been shown to protect mammalian cells, including cancer cells, against oxidative stress. Proline protection has been postulated to involve protein chaperoning, direct scavenging of ROS, and the upregulation of antioxidant enzymes [14, 15]. Our experimental results show that ORAOV1 binds to PYCR1 and PYCR2, that cell survival after stress treatment was significantly higher in *ORAOV1*-overexpressed cell lines than in controls, and that the stress-resistant effect was cancelled by *PYCR*-knockdown. Furthermore, after stress treatment, the intracellular proline concentrations were significantly higher and ROS production was lower in an *ORAOV1*-overexpressed cell line than in the control. These findings indicate that ORAOV1 influences PYCR activity, and as a result, the intracellular proline level increases and ROS production is reduced, thereby promoting tumor progression (Figure 7B). Therefore, as was seen in our study, the *ORAOV1* gene enhances tumorigenicity and tumor growth.

In our cohort, the *ORAOV1* gene was amplified in approximately half of the stage III ESCC, and patients with *ORAOV1* amplification tended to have a shorter OS than patients without amplification, although the difference was not significant. Our experimental findings indicate that the *ORAOV1* gene enhances tumorigenicity and tumor growth and is associated with a poorly differentiated histology among ESCCs. Thus, these factors might be correlated with a poor prognosis. Although a previous study has shown that *ORAOV1* overexpression is significantly associated with lymph node metastasis and advanced TNM stages in ESCC [11], our study is the first report to suggest a possible association with a poor prognosis. However, the *ORAOV1* gene was identified within chromosomal band 11q13, which includes several known cancer-related genes such as *CCND1*, *FGF4*, *FGF3*, and *CTTN* [6]. Therefore, patients with *ORAOV1* amplification may exhibit the amplification of other cancer-related genes, which might also be related to a poor prognosis.

*ORAOV1* amplification was significantly associated with a poorly differentiated histology and, in our xenograft study, *ORAOV1*-overexpressed cells produced poorly differentiated tumors. The *ORAOV1* gene was first identified in oral squamous cell cancer and is frequently amplified, and some previous studies have shown similar results for oral squamous cell cancer [7, 19-21]. Recent studies have suggested that ROS is required for differentiation [22]. Therefore, the *ORAOV1* gene might be related to poorly differentiated tumors via a reduction in ROS production.

In conclusion, the *ORAOV1* gene is frequently amplified in ESCC, enhances tumorigenicity and tumor growth, and is associated with a poorly differentiated histology via proline metabolism and ROS production. Our findings indicate that ORAOV1 might be a novel target for the treatment of ESCC.

## MATERIALS AND METHODS

### Cell culture

The HEK293 cell line (human embryonic kidney cell line) was maintained in DMEM medium (Nissui Pharmaceutical, Tokyo, Japan) supplemented with 10% FBS (GIBCO BRL, Grand Island, NY). The KYSE70 cell line (human ESCC cell line) was maintained in DMEM medium with 2% FBS. The KYSE170 and KYSE220 cell lines (human ESCC cell lines) were maintained in a 1:1 mixture of RPMI1640 and Ham's F12 medium (Sigma-Aldrich, St. Louis, MO) with 2% FBS. The T.T cell line (human ESCC cell line) was maintained in a 1:1 mixture of DMEM and Ham's F12 medium with 10% FBS. All the cell lines were maintained in a 5% CO_2_-humidified atmosphere at 37°C.

### Real-time RT-PCR

One microgram of total RNA from normal tissue purchased from Clontech (Palo Alt, CA)) and BioChain (Newark, CA) and from cultured cell lines was converted to cDNA using the GeneAmp RNA-PCR kit (Applied Biosystems, Foster City, CA). Real-time PCR was performed using the Applied Biosystems 7900HT Fast Real-time PCR System (Applied Biosystems), as described previously [23]. The glyceraldehyde 3-phosphate dehydrogenase (*GAPD*, NM_002046) gene was used to normalize the expression levels in subsequent quantitative analyses. To amplify the target genes, the following primers were used: *ORAOV1*-F1, GCTGGCAGTCAGGACATATTCG; *ORAOV1*-R1, CTGCCTTCCCTCCATCACAC; *GAPD*-F, GCACCGTCAAGGCTGAGAAC; *GAPD*-R, ATGGTGGTGAAGACGCCAGT.

### Patients

The criteria for eligibility were histologically confirmed ESCC, surgery for stage III disease without prior radiotherapy or chemotherapy before surgery, and the availability of an FFPE sample. Tumor specimens were collected from 94 patients with ESCC who were treated at the Kinki University Faculty of Medicine between 2003 and 2010. The World Health Organization Classification of Tumors was used for histological grading. The tumors were staged according to the TNM classification of the American Joint Committee on Cancer (AJCC)/Union for International Cancer Control (UICC). This study was retrospectively performed and approved by the institutional review board of the Kinki University Faculty of Medicine.

### Isolation of genomic DNA

Genomic DNA samples were extracted from surgical specimens preserved as FFPE tissue using the QIAamp DNA Micro kit (Qiagen, Hilden, Germany), according to the manufacturer's instructions as described previously [24]. Macro-dissection of the FFPE samples was performed to select a cancer region, which was marked by a pathologist after deparaffinization. The DNA concentration was determined using the NanoDrop2000 (Thermo Fisher Scientific, Waltham, MA).

### Copy number assay for *ORAOV1* genes

The copy numbers for the *ORAOV1* gene were determined using commercially available and pre-designed TaqMan Copy Number Assays, according to the manufacturer's instructions (Applied Biosystems) as described previously [24]. The primer IDs used for the *ORAOV1* gene were Hs03772057_cn (intron 2) and Hs03793932_cn (intron 3). The *TERT* gene locus was used for the internal reference copy number. Human Genomic DNA (TaKaRa, Shiga, Japan) was used as a normal control. The resulting products were detected using the ABI PRISM 7900 HT Sequence Detection System (Applied Biosystems). Data were analyzed using SDS 2.2 software and CopyCaller software (Applied Biosystems).

### Expression vector construction and viral production

Expression vector construction and viral production were performed as previously described [23]. Briefly, a full-length cDNA fragment encoding the human *ORAOV1* gene was obtained from KYSE170 using RT-PCR, and the *ORAOV1* gene was isolated using PCR and Prime STAR HS DNA polymerase (TaKaRa) with the following primers: *ORAOV1*-F2, GCCGCCATGGCTGGCAGTCAGGACATA; *ORAOV1*-R2, GCCCAGTCAAAATGAAAGTCCGGAACC. The sequences of the PCR-amplified DNAs were confirmed by sequencing after cloning into a pCR-Blunt II-TOPO cloning vector (Invitrogen, Carlsbad, CA). *ORAOV1* cDNA in the TOPO cloning vector was cut out and transferred to a pQCLIN retroviral vector (Clontech) together with enhanced green fluorescent protein (EGFP) following the internal ribosome entry site sequence (IRES) to monitor the expression of the inserts indirectly. A pVSV-G vector (Clontech) for the constitution of the viral envelope and the pQCLIN-IG constructs were cotransfected into gpIRES-293 cells using FuGENE6 transfection reagent (Roche Diagnostics, Basel, Switzerland). After 48 hours of transfection, the culture medium was collected and the viral particles were concentrated by centrifugation at 15,000 x*g* for 3 hours at 4°C. The viral pellet was then resuspended in fresh DMEM medium. The titer of the viral vector was calculated by counting the EGFP-positive cells that were infected by serial dilutions of virus-containing media, and the multiplicity of infection was then determined. The viral vector and stable viral transfectant cell lines were designated as pQCLIN-EGFP, pQCLIN-ORAOV1, KYSE70-pQCLIN-EGFP, KYSE70-pQCLIN-ORAOV1, KYSE170-pQCLIN-EGFP, and KYSE170-pQCLIN-ORAOV1.

To remove the stop codon (TGA) from human *ORAOV1* cDNA and to construct the ORAOV1/HA/His fusion protein, the *ORAOV1* gene was again isolated from the cDNA using the following primers: *ORAOV1*-F3, GCCGCCATGGCTGGCAGTCAGGACATA; *ORAOV1*-R3, TGCAAATGAAAGTCCGGAACCTTCTGC. After the sequences were confirmed using the pCR-Blunt II-TOPO cloning vector, *ORAOV1* cDNA (without a stop codon) was fused to a HA/His-containing pcDNA3.1 vector (Clontech). The ORAOV1/HA/His vector was then transfected into the HEK293 cell line using FuGENE6 transfection reagent. Hygromycin selection (100 μg/mL) was performed on days 2-8 after transfection, and the cells were then cultured in normal medium. The stable transfectant HEK293 cell line was designated as HEK293-pcDNA-ORAOV1/HA/His.

### Antibody

A rabbit antibody specific for ORAOV1 was obtained from Abcam (Cambridge, United Kingdom). A rat high-affinity antibody specific for HA was obtained from Roche Diagnostics. A mouse antibody specific for PYCR1 and a rabbit antibody specific for PYCR2 were obtained from Sigma-Aldrich and Abnova, respectively. A rabbit antibody specific for β-actin was obtained from Cell Signaling (Beverly, MA).

### Western blot analysis

A western blot analysis was performed as described previously [23]. Briefly, subconfluent cells were washed with cold phosphate-buffered saline (PBS) and harvested with Lysis A buffer containing 1%Triton X-100, 20 mM Tris-HCl (pH7.0), 5 mM EDTA, 50 mM sodium chloride, 10 mM sodium pyrophosphate, 50 mM sodium fluoride, 1 mM sodium orthovanadate and a protease inhibitor mix, Complete™ (Roche Diagnostics). Whole-cell lyses were separated using SDS-PAGE and were blotted onto a polyvinylidene fluoride membrane. After blocking with 3% bovine serum albumin in a TBS buffer (pH8.0) with 0.1% Tween-20, the membrane was probed with primary antibody. After rinsing twice with TBS buffer, the membrane was incubated with horseradish peroxidase-conjugated secondary antibody and washed, followed by visualization using an ECL detection system (GE Healthcare, Buckinghamshire, United Kingdom) and LAS-3000 (Fujifilm, Tokyo, Japan).

### Cellular growth assay

The KYSE70 and KYSE170 transfectant cell lines were incubated on 96-well plates at a density of 2,000/well with 200 μL of cultured medium at 37°C in 5% CO_2_. After 24, 48, or 72 hours of incubation, 20 μL of MTT [3-(4,5-dimethyl-thiazoyl-2-yl) 2,5-diphenyltetrazolium bromide] solution (Sigma-Aldrich) was added, the culture medium was discarded, and the wells were filled with DMSO. The absorbance of the cultures at 570 nm was measured using VERSAmax (Japan Molecular Devices, Tokyo, Japan). The average O.D. values of the 6 wells were used for a single experiment, and the experiment was performed in triplicate.

### Colony formation assay

The KYSE70 and KYSE170 transfectant cell lines were seeded into 6-well plates containing 1,000 cells/well. The medium was exchanged every 4 days. After 2 weeks, the cells were washed with PBS and were fixed with 4% paraformaldehyde for 10 min, then stained with 0.1% crystal violet for 15 min; the colonies were then counted under a light microscope (IX71; Olympus, Tokyo, Japan). The experiment was performed in triplicate.

### Cellular adhesion assay

A 96-well plate was coated with solid gel basement membrane proteins Matrigel (BD Biosciences, Franklin Lakes, NJ) or fibronectin (BD Biosciences). The KYSE70 and KYSE170 transfectant cell lines (20,000 cells /well) were added to the wells of coated plates and incubated at 37°C for 1 hour. The wells were then washed twice with PBS to remove nonadherent cells. Adherent cells were evaluated using the MTT assay, as described above. The average O.D. values of 6 wells were used for a single experiment, and the experiment was performed in triplicate.

### Migration assay

The migration assays were done as described previously [23] using the Boyden chamber methods and polycarbonate membranes with an 8-μm pore size (Chemotaxicell; KURABO, Osaka, Japan). The membranes were coated with fibronectin on the outer side and were dried for 2 hours at room temperature. The KYSE70 and KYSE170 transfectant cell lines (20,000/well) were then seeded into the upper chambers with 200 μL of 0.5% FBS migration medium, and the upper chambers were placed into the lower chambers of 24-well culture dishes containing 600 μL of cultured medium. After incubation for 24 hours, the media in the upper chambers were aspirated and nonmigrated cells on the inner sides of the membranes were removed using a cotton swab. The cells that had migrated to the outer side of the membrane were fixed with 4% paraformaldehyde for 10 min, stained with 0.1% crystal violet for 15 min, and then counted using a light microscope. The number of migrated cells was averaged using 5 fields per one chamber, and three chambers were used for one experiment. The experiment was done in triplicate.

### Scratch assay

The KYSE70 and KYSE170 transfectant cell lines were plated onto 6-well plates and were incubated until they reached confluence. Wounds were introduced to the confluent cell monolayer using a plastic pipette tip. The cells were then incubated at 37°C. After 12 hours, the scratch area was photographed using a light microscope. The wound distance from edge to edge was measured and was averaged using 5 points per 1 wound area. Three wound areas were evaluated in one experiment, and the experiment was performed in triplicate.

### Xenograft studies

Nude mice (BALB/c nu/nu; 6-week-old females; CLEA Japan, Tokyo, Japan) were used for the *in vivo* studies and were cared for in accordance with the recommendations for the Handling of Laboratory Animals for Biomedical Research compiled by the Committee on Safety and Ethical Handling Regulations for Laboratory Animals Experiments, Kinki University. The ethical procedures followed and met the requirements of the United Kingdom Coordinating Committee on Cancer Research guidelines [25]. To evaluate tumorigenicity, a suspension of 1 × 10^6^ KYSE70 transfectant cells (in 50 μL PBS) were subcutaneously inoculated into both flanks of nude mice (n = 8). To evaluate the tumor growth, a suspension of 5 × 10^6^ KYSE70 transfectant cells (in 50 μL PBS) with 50 μL of Matrigel were subcutaneously inoculated into nude mice (n = 5). The tumor volume was calculated as the length × width^2^ × 0.5. The tumor formation and volume were assessed every 2 to 3 days. At the end of the experiment, the mice were sacrificed and the xenografts were resected, fixed in 10% buffered formalin for 6 to 10 hours, and processed for histologic analysis. The method was described previously [26].

### Construction of *E. coli* expression vectors with the *ORAOV1* gene and purification of fusion protein from *E. coli*

To insert *ORAOV1* into a pMAL-c2X vector (New England Biolabs, Beverly, MA), the *ORAOV1* gene was again isolated from cDNA using the following primers: *ORAOV1*-F4, GCCGCCATGGCTGGCAGTCAGGACATA; *ORAOV1*-R4, GCTTAAATGAAAGTCCGGAACCTTCTGC. After the sequences were confirmed using the pCR-Blunt II-TOPO cloning vector, *ORAOV1* cDNA was fused to a pMAL-c2X vector downstream of the *malE* gene, which encodes MBP. The vectors and fusion proteins were designated as pMAL-mock, pMAL-ORAOV1, MBP-mock, and MBP-ORAOV1, respectively.

Competent *E. coli* cells were transformed with pMAL-mock and pMAL-ORAOV1. Selected positive clones were cultured at 37°C for 3 hours and induced using 0.5 mM isopropyl-β-D-thiogalactopyranoside (IPTG; Sigma-Aldrich) for 3 hours at 37°C. After induction with IPTG, the *E. coli* cells were diluted and treated ultrasonically at 4°C in an ice water bath, and the debris was then removed by centrifugation (9,000 x*g* for 10 min at 4°C). To absorb the protein, the sample was incubated with amylose resin (New England Biolabs) for 1 hour at 4°C. After incubation, the absorbed resin was applied to a polystyrene column (Thermo Fisher Scientific). MBP-mock or MBP-ORAOV1 was eluted from the column using 10 mM of maltose.

### Analysis of binding protein with MBP-ORAOV1

Cell lyses of the T.T or KYSE220 cell line (5 mg) were mixed with 100 μg of MBP-mock or MBP-ORAOV1 for 2 hours at 4°C. To absorb MBP-mock or MBP-ORAOV1with binding protein, the samples were incubated with amylose resin for 1 hour at 4°C. After incubation for 5 min at 98°C, the samples were separated using SDS-PAGE and the gel was stained using the silver stain method. Silver-stained protein bands of interest were cut out from the SDS-PAGE gel and the proteins were digested with trypsin (Proteomics grade, Sigma-Aldrich) overnight at 37°C. The mass spectrometry findings were acquired using a matrix-assisted laser desorption ionization/time-of-flight mass spectrometer (Voyager-DE STR; Applied Biosystems). Protein identification for the generated data was performed by searching a database using the online MASCOT search engine.

### Immunoprecipitation and immunoblotting analysis

Cell lyses of the HEK293-pcDNA-ORAOV1/HA/His cell line (1 mg) were incubated with normal rat IgG (Santa Cruz Biotechnology, Santa Cruz, CA) or anti-HA high affinity antibody for immunoprecipitation. Immunocomplexes were purified by incubation with protein G-agarose beads (Santa Cruz Biotechnology), separated using SDS-PAGE after incubation for 5 min at 98°C, and then blotted onto a polyvinylidene fluoride membrane. The subsequent immunoblotting analysis was performed as described above.

### Cell survival after stress treatment

For stress treatment, the KYSE70 and KYSE170 transfectant cell lines were plated onto 96-well plates at a density of 20,000/well with FBS 0.5% medium at 37°C in 5% CO_2_. After 24 hours of incubation, the cells were treated for 12 hours with 50 μM H_2_O_2_. Cell survival after stress treatment was estimated using the MTT assay as described above. The average O.D. values of the 6 wells were used for a single experiment, and the experiment was performed in triplicate.

### Short interfering RNA (siRNA) transfection

Cells were transfected with siRNA for PYCR1 and 2 or each non-specific target (scramble) as follows: CAGUUUCUGCUCUCUCAGGAA for PYCR1, GUGAUUCGCUGCAUGACCA for PYCR2, GCUCUGUCCGUUAAUCGAA for scramble of PYCR1, and AAUUAUGCGCGUAUCCGCG for scramble of PYCR2. siRNA transfection was performed using RNAiMAX (Invitrogen, Carlsbad, CA) according to the manufacturer's instructions, as previously described [26], and was allowed to proceed for 48-96 hours before the stress test or the collection of the whole-cell extract. Knockdown was confirmed using western blot analyses.

### Intracellular proline concentrations and ROS measurement

To measure the intracellular proline concentrations, we used KYSE70 transfectant cell lines. The culture medium was replaced with 0.5% FBS medium with or without 50 μM H_2_O_2_. Then, after 12 hours of incubation, each of the cell lines (2 × 10^7^ cells) was washed twice with PBS and pelleted using centrifugation. The cell pellets were lysed with 240 μL of methanol and were treated with ultrasonic. Debris was removed by centrifugation, and the proline concentrations were determined using high-performance liquid chromatography fluorescence detection and the pre-column derivatization procedure, as previously described [27]. The experiment was performed in triplicate.

To measure ROS, the KYSE70 transfectant cell lines were plated onto a 6-cm dish at a density of 1 × 10^6^/dish at 37°C in 5% CO_2_. After 24 hours of incubation, the culture medium was replaced with 0.5% FBS medium and the cells were treated for 12 hours with or without 50 μM of H_2_O_2_. Then, CellROX Deep Red Reagent (Invitrogen) was added at a final concentration of 5 μM. After 30 min of incubation at 37°C, the cells were harvested by trypsinization and washed three times with PBS. The cells were analyzed using a flow cytometer (BD FACSCalibur™; BD Biosciences). The experiment was performed in triplicate.

### Statistical analysis

Continuous variables were analyzed using the Student t-test, and the results were expressed as the average and standard deviations (SD). Dichotomous variables were analyzed using the Fisher exact test. Disease-free survival (DFS) was defined as the time from the surgery until the first observation of disease progression or death from any cause, and the overall survival (OS) was defined as the time from the surgery until death from any cause. The DFS and OS curves were constructed using the Kaplan-Meier method and were compared among groups using the log-rank test. The statistical analyses were two-tailed and were performed using Microsoft Excel (Microsoft, Redmond, WA). A *P*-value of less than 0.05 was considered statistically significant.

## SUPPLEMENTARY FIGURE


