# Supplementary material for: Frequent amplification of ORAOV1 gene in esophageal squamous cell cancer promotes an aggressive phenotype via proline metabolism and ROS production
Source: Oncotarget. 2013 Dec 30;5(10):2962–73. doi: 10.18632/oncotarget.1561 (PMC4102783; doi:10.18632/oncotarget.1561)
Supplement: Supplementary file 1 [file oncotarget-05-2962-s001.pdf]

Frequent amplification of *ORAOV1* gene in esophageal squamous cell cancer promotes an aggressive phenotype via proline metabolism and ROS production-Togashi et al

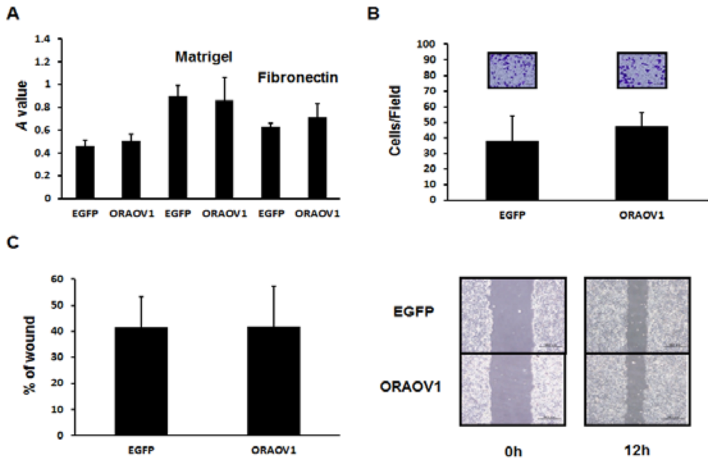

Supplementary Figure 1: The *ORAOV1* gene was not associated with cellular motility. A, Adhesion assay in KYSE170 transfectant cell lines. A 96-well plate was coated with Matrigel or fibronectin. The KYSE170 transfectant cell lines (20,000 cells/well) were added to the wells of the coated plates and were incubated for 1 hour. The wells were then washed to remove nonadherent cells. Adherent cells were evaluated using an MTT assay. The experiment was performed in triplicate. No difference was observed between the KYSE70-pQCLIN-EGFP and the KYSE170-pQCLIN-ORAOV1cell lines (non-coated, P = 0.44, Matrigel, 0.73 and fibronectin, 0.42, respectively). B, Migration assay in KYSE170 transfectant cell lines. The migration assays were performed using the Boyden chamber method. The membranes were coated with fibronectin on the outer side. The KYSE170 transfectant cell lines (20,000 cells/well) were then seeded into the upper chambers. After incubation for 24 hours, nonmigrated cells on the inner sides of the membranes were removed, and the cells that had migrated to the outer side of the membrane were stained with crystal violet, then counted using a light microscope. The experiment was done in triplicate. There was no difference in the migration cell counts between the KYSE70-pQCLIN-EGFP and the KYSE170-pQCLIN-ORAOV1 cell lines (37.53 ± 16.59 vs. 47.02 ± 9.21 cells/field, P = 0.16). C, Scratch assay in KYSE170 transfectant cell lines. The KYSE170 transfectant cell lines were plated onto 6-well plates and were incubated until they reached confluence. Wounds were introduced to the confluent cell monolayer using a plastic pipette tip. After incubation for 12 hours, the wound distance from edge to edge was measured. The experiment was performed in triplicate. There was no difference in wound closure between the KYSE70-pQCLIN-EGFP and the KYSE170-pQCLIN-ORAOV1 cell lines (41.46% ± 11.91% vs. 41.83% ± 15.50%, P = 0.98).
